# A self-managed exercise therapy program for wrist osteoarthritis: study protocol for a randomized controlled trial

**DOI:** 10.1186/s13063-023-07668-4

**Published:** 2023-10-02

**Authors:** Sara L. Larsson, Elisabeth Ekstrand, Lars B. Dahlin, Anders Björkman, Elisabeth Brogren

**Affiliations:** 1 Department of Hand Surgery, Skåne University Hospital, Malmö, Sweden; 2 Department of Translational Medicine - Hand Surgery, Lund University, Jan Waldenströms Gata 5, 205 03 Malmö, Sweden; 3 Department of Health Sciences, Lund University, Lund, Sweden; 4 Department of Biomedical and Clinical Sciences, Linköping University, Linköping, Sweden; 5 Department of Hand Surgery, Institute of Clinical Sciences, Sahlgrenska University Hospital, Sahlgrenska Academy, University of Gothenburg, Gothenburg, Sweden

**Keywords:** Wrist osteoarthritis, SLAC, SNAC, Exercise therapy, Neuromuscular control, Self-management, Randomized controlled trial

## Abstract

**Background:**

Post-traumatic wrist osteoarthritis (OA) can eventually lead to pain, muscular weakness, and stiffness of the wrist, which can affect the function of the entire upper limb and reduce the quality of life. Although there is strong evidence that all patients with OA should be offered adequate education and exercises as a first-line treatment, an effective self-management program, including structured education and therapeutic exercises, has not yet been introduced for individuals with wrist OA. This trial aims to evaluate the effectiveness of an exercise therapy program with joint protective strategies to improve neuromuscular control (intervention group) compared to a training program with range of motion exercises (control group).

**Methods:**

This is a single-blinded randomized controlled trial (RCT) with two treatment arms in patients with symptomatic and radiographically confirmed wrist OA. The trial will be conducted at a hand surgery department. The participants will be randomly assigned either to a neuromuscular exercise therapy program or to a training program with range of motion exercises only. Participants in both groups will receive a wrist orthosis and structured education on wrist anatomy, pathophysiology, and joint protective self-management strategies. The programs consist of home exercises that will be performed twice a day for 12 weeks. The Patient-Rated Wrist Evaluation (PRWE) is the primary outcome measure of pain and function. Wrist range of motion (ROM), grip strength, the Numeric Pain Rating scale (NPRS), Disabilities of the Arm, Shoulder, and Hand (DASH), the General Self-Efficacy Scale (GSES), Global Rating of Change (GROC), and conversion to surgery are the secondary measures of outcome. Assessments will be performed at baseline and at 3, 6, and 12 months after baseline by a blinded assessor.

**Discussion:**

The upcoming results from this trial may add new knowledge about the effectiveness of a self-managed exercise therapy program on pain and function for individuals with wrist OA. If the present self-management program proves to be effective, it can redefine current treatment strategies and may be implemented in wrist OA treatment protocols.

**Trial registration:**

ClinicalTrials.gov, NCT05367817. Retrospectively registered on 27 April 2022. https://clinicaltrials.gov.

**Supplementary Information:**

The online version contains supplementary material available at 10.1186/s13063-023-07668-4.

## Background

The wrist joint performs complex movements in multiple directions and sustains high loads in many positions. Its involvement in most daily tasks, with great demands on mobility, stability, and load-bearing forces, puts the wrist at risk for problems after injuries and degenerative diseases, such as osteoarthritis (OA) [1]. The prevalence of wrist OA is low but increases with age [2]. In contrast to OA in other joints of the hand, wrist OA develops earlier in life and is more common in men (prevalence 1.7%) than in women (1.0%) [2, 3].

The cause of wrist OA is usually secondary due to a previous traumatic insult, such as a fracture or ligament injury. It selectively involves the joints surrounding the scaphoid bone [4]. Two common types of wrist OA patterns are the scaphoid non-union advanced collapse (SNAC), which is instigated by an unhealed fracture of the scaphoid [5], and the scapholunate advanced collapse (SLAC), which is caused by a traumatic or degenerative scapholunate ligament injury [6]. The SNAC and SLAC cause carpal instability, altered wrist kinematics, and joint loading with eventual arthritic degeneration of the radiocarpal and midcarpal joints [4]. A four-staged predictable and progressive pattern ranges from stage 1 that represents mild arthritic changes confined to the radial styloid to stage 4 that represents advanced arthritic changes affecting both the radiocarpal and midcarpal joints [6].

Post-traumatic wrist OA develops slowly, and the joint degeneration can lead to pain, muscular weakness, and stiffness of the wrist [7]. As a result, this can affect the function of the entire upper limb, which can interfere with activities of daily living (ADL) and the ability to work, thus leading to reduced quality of life [8]. In an OA affected joint, disturbed neuromuscular control can lead to a disproportionate load on the joint [9]. This unhealthy progress could aggravate the OA progression over time [10].

Currently, the treatment norm for wrist OA is initially directed at alleviating pain and decrease disability by splinting, non-steroidal anti-inflammatory medications, and intraarticular steroid injections. The surgical interventions for wrist OA include neurectomy, styloidectomy, proximal row carpectomy, fusions, or arthroplasty [11]. However, a self-managing approach, including therapeutic exercises, has traditionally not been a treatment strategy in wrist OA.

Exercise therapy is a regime of physical activities designed and prescribed for precise therapeutic goals, aiming at educating the performance of specific exercises to improve neuromuscular control, reduce pain, and achieve functional joint stability [12]. Self-management programs, including exercise therapy and joint protective strategies, are core treatments in knee and hip OA [13–17]. Due to the complexity of the wrist joint, it cannot be fully compared to larger weight-bearing joints, such as the knee and hip. Therefore, there is a need to develop exercise therapy programs specially designed for the wrist. Such a program should be part of a comprehensive joint protective standard care and could be beneficial to decrease disability and postpone, or possibly even eliminate, the need for surgery in individuals with wrist OA.

The objective of the trial is to evaluate the effectiveness of an exercise therapy program with joint protective strategies to improve neuromuscular control (intervention group) compared to a training program with range of motion (ROM) exercises (control group). To evaluate these programs, we will use the Patient-Rated Wrist Evaluation (PRWE) as our primary outcome [18]. We hypothesize that 12 weeks of exercise therapy relieves pain and improves the ability to perform daily activities to a greater extent than the ROM training program.

## Methods/design

### Trial design

**Fig. 1:**
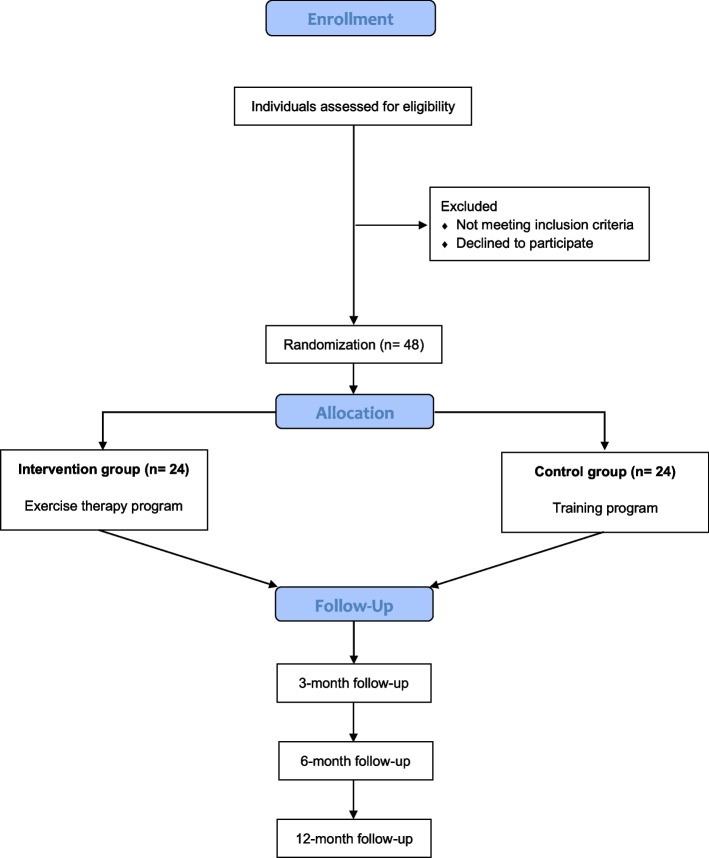
Flowchart of the trial design

**Fig. 2:**
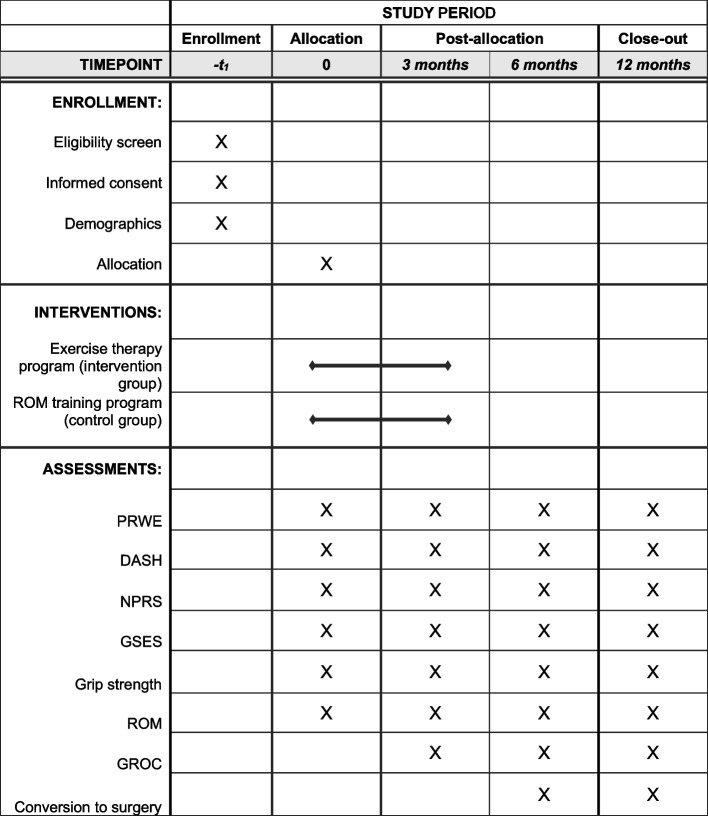
SPIRIT diagram of enrollment, interventions, and outcome measures. SPIRIT, The Standard Protocol Items: Recommendations for Interventional Trials; PRWE, Patient-Rated Wrist Evaluation; DASH, Disabilities of the Arm, Shoulder, and Hand; NPRS, Numerical Pain Rating Scale; GSES, Generalized Self-Efficacy Scale; ROM, range of motion; GROC, Global Rating of Change

This RCT utilizes a single-blinded (assessor) superiority trial design with two treatment arms. The Standard Protocol Items: Recommendations for Interventional Trials (SPIRIT) checklist [19] will be used and is provided as an Additional file 1. Forty-eight individuals with radiographically confirmed and symptomatic wrist OA will be recruited and randomized either to the neuromuscular exercise therapy program (*n* = 24) or to the ROM training program (*n* = 24). A description of the overall trial design can be found in the flow chart (Fig. 1) and the SPIRIT diagram (Fig. 2).

### Sample size calculation

The sample size estimate of this RCT is based on relevant previous studies that have shown minimal clinically important differences (MCIDs) for the PRWE between 11.5 and 14 [20, 21]. An MCID of 11.5 has been found in patients with distal radius fractures [20], while an MCID of 14 was found in patients with various atraumatic upper extremity disorders, including patients with wrist OA [21]. We have calculated our sample size of an MCID in between these two studies (MCID 12.5). Using a standard deviation (SD) of 14, power (1-beta) at 0.8, and a significance level (alpha) at 0.05, we will need to recruit a sample of 40 patients, 20 in each group. Accounting for a drop-out rate of 20%, we will ultimately need to include a total of 48 patients in the trial (Fig. 1). We will continue to recruit participants until we have reached our estimated sample size. If a participant withdraws from the trial before completing the 12-week allocated treatment program, we will recruit a new participant. Participants that withdraw from the trial between the 3- and 12-month follow-ups will not be replaced.

### Eligibility criteria

Participants will be selected according to the following eligibility criteria:

Inclusion criteria:

1. Radiographically confirmed and symptomatic wrist OA—SLAC and SNAC stages 1–3 [6]
2. Age ≥ 18

Exclusion criteria:

1. The presence of other diseases or disorders that could affect arm and hand function
2. Wrist osteoarthritis secondary to avascular necrosis of carpal bones
3. Previous surgery to the wrist
4. Intraarticular wrist cortisone injection within the last 3 months
5. Inability to understand and follow test instructions due to communicative, mental, or cognitive impairments

### Study setting and inclusion of participants

Potential participants will be identified and recruited at the Department of Hand Surgery, Skåne University Hospital, Malmö, Sweden. The study center is the main health care facility to which individuals with wrist OA are referred in the Southern health care region; a region with approximately 1.9 million inhabitants.

When individuals with wrist OA are referred to our tertiary hand surgery clinic, their symptoms are usually advanced and affecting everyday life to the extent that they want to discuss treatment options with a hand surgeon. For this RCT, we will implement a new routine. Before deciding on surgical treatment, potential participants, diagnosed with wrist OA stages 1–3, will be referred to one treating physiotherapist (PT) who is a specialist in treating orthopedic injuries and with long experience working at the hand surgery clinic. They will be provided with all the relevant information about the trial, verbally and in writing, and be asked for participation. They will be told that they will be randomly assigned to the trial groups and that each group will be treated with different types of exercises, all of which are appropriate for their condition. The treating PT will be responsible for the treatment programs in both groups, including structured education, exercises, and follow-ups.

### Ethical aspects

Prior to inclusion, information about the trial will be provided, and the participants will give their written informed consent to participate (Additional file 2). This study was approved by the Swedish Ethical Review Authority, Dnr 2019–02437, and the principles of the Declaration of Helsinki will be followed.

### Randomization and allocation

An experienced occupational therapist (OT), who is also a researcher at the hand surgery clinic, will generate the block randomization sequence. This person will be independent of the research team responsible for data collection and management. The treating PT will create the sealed paper envelopes, but the independent OT will generate the allocation sequence. The sequence will be generated using block randomization with the size of 10 in each block. The block randomization intends to achieve balance for the distribution of men and women to be similar in the two groups over time, along with other known factors that could affect the outcome, such as age and severity of the OA. The treating PT will allocate the participants by sealed paper envelopes either to the exercise therapy program (intervention group, *n* = 24) or to the ROM training program (control group, *n* = 24) on the same day as the baseline assessment (Fig. 1). Randomization codes will be generated digitally and concealed on a secure system.

### Blinding

Participants will not be directly informed about which group they have been assigned to, which limits the risk of contamination between the groups. To further avoid the risk of contamination between the groups, the same treating PT will be responsible for giving out the allocated treatment regimens and seeing the participants at all the follow-up appointments. Also, the participants will be booked to the clinic on an individual basis, which means that they will not meet each other at the clinic.

All the objective and subjective evaluations will be conducted by one blinded PT with experience of the used outcome measures. Apart from the blinded PT, the treating hand surgeons and the hand surgeons assessing the radiological wrist OA stage will also be blinded to group allocation. The blinding will be maintained as far as possible. The actual allocation should not be disclosed to the participant and/or other trial personnel nor should there be any written or verbal disclosure of the code in any of the corresponding patient documents. We do not anticipate any safety concerns of the participants in both groups that would lead to emergency unblinding. The only situation in which unblinding could occur, and would be permissible, is if a participant wants to know their group allocation. If this unblinding occurs, the participant will be excluded from further participation in the trial.

### Baseline assessment

At the baseline assessment, background information will be collected concerning (1) medical and social history (civil status, occupation, sports, hobbies); (2) demographic data (age, gender, handedness, review of the nature and onset of symptoms); (3) use of painkillers; and (4) previous exercise treatment (physiotherapy or occupational therapy). The participants will also, in pre-defined answer options using a box scale, report (1) their main problem with the wrist (answer options: pain, weakness, stiffness); (2) their main expectation of the exercise program (answer options: reduced pain, improved strength, improved ROM); (3) if they have discussed surgical treatment with their treating hand surgeon (yes/no), and if so, which type of surgery; and (4) their attitude towards proceeding with surgery (yes/no).

### Imaging and classification of OA

Potential participants with wrist OA seeking care at our hand surgery clinic are examined with standard wrist radiographs in posterior-anterior and lateral views. Radiological diagnosis, in combination with clinical examination by the treating hand surgeon, confirms the diagnosis of symptomatic wrist OA. Participants meeting the inclusion criteria will also be examined with computer tomography (CT) of the affected wrist to enable a detailed examination of osteoarthritic signs.

To grade the severity of wrist OA, the modified four-stage Watson and Ballet classification of SLAC and SNAC will be adopted [6]. The classification contains stage 1 (arthritic changes confined to the radial styloid), stage 2 (arthritic changes between the radius and the entire scaphoid), stage 3 (in addition to grades 1 and 2, arthritic changes at the capitate-lunate joint), and stage 4 (in addition to grade 3, arthritic changes in the radio-lunate fossa) [7]. Two experienced hand surgeons will independently classify OA based on both plain radiographs and CT scans of the affected wrist. In cases of disagreement between the observers, consensus will be reached through discussions.

## Interventions

### Procedure

The participants will be provided with the allocated exercise therapy (intervention group) or ROM training program (control group) on the same day as their baseline assessment. They will be taught how to perform their exercises in a pain-free range with good quality of movement—smooth, coordinated, and without compensatory movements [22]. The treatment will then continue as a structured home-based program that the participants will perform twice a day for 12 weeks. All participants will receive a booklet including structured education and a description of the exercises with schematic images. The participants will also be offered a follow-up appointment with their treating hand surgeon between 3 and 6 months after baseline to decide if further treatment or conversion to surgery is needed.

## Concomitant care

While the trial is in progress, no other treatments will be allowed or prescribed. The information provided to participants will not specify any prohibitions. Participants will be able to take their usual pain medication, such as paracetamol or non-steroidal anti-inflammatory drugs (NSAIDs), if needed. However, they will not be subscribed pain medication, such as opioids, or intraarticular wrist cortisone injection during their participation in the trial.

### Structured education

**Fig. 3:**
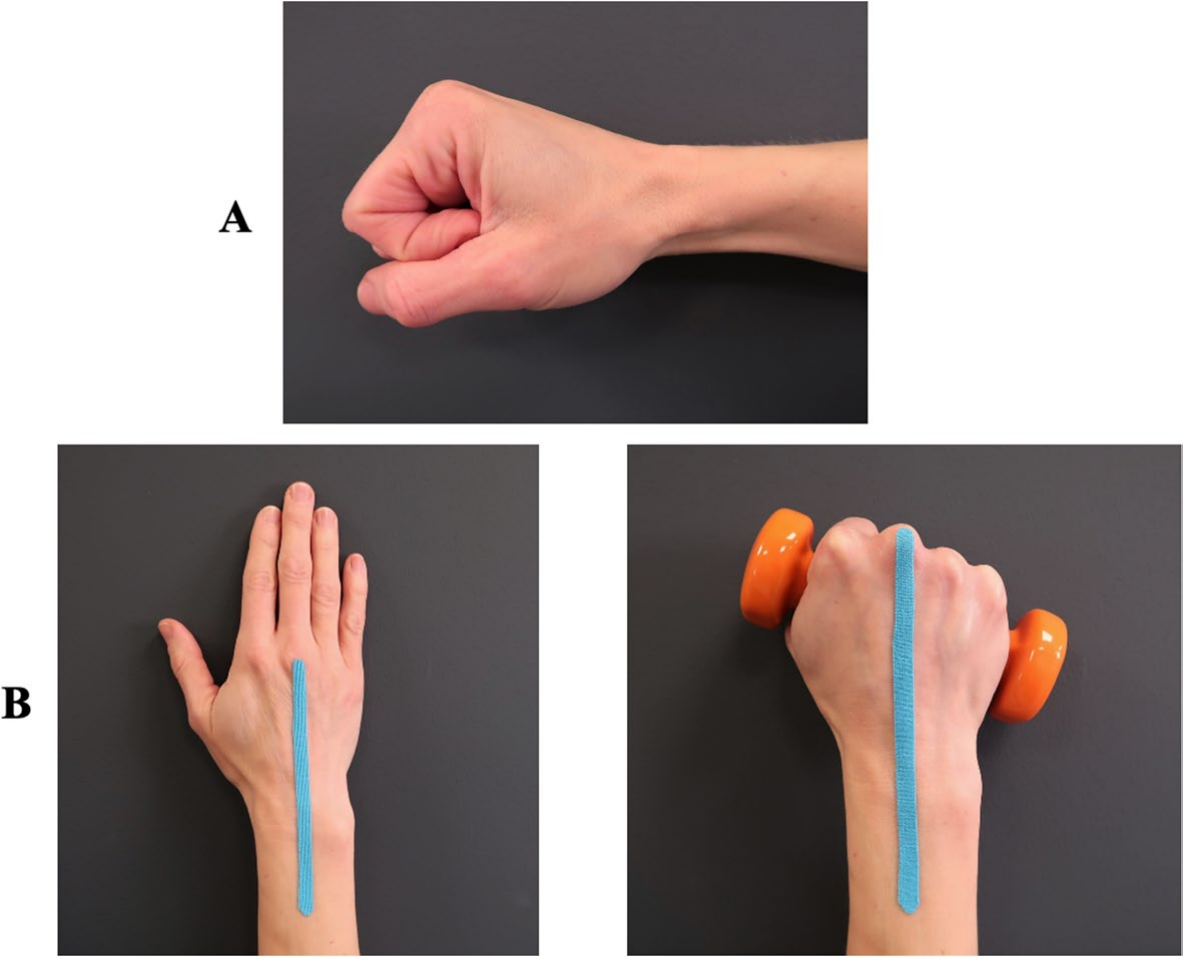
The neutral wrist position. **A** In the sagittal plane (lateral view), the wrist is in slight extension. **B** In the frontal plane, the third metacarpal bone is in line with the forearm

The participants in both groups will receive structured education on (1) wrist anatomy, (2) pathophysiology, (3) joint protective self-management strategies, and (4) management of pain and fatigue by exercises (Table 1). They will also be educated about the neutral wrist position, which is in slight extension of approximately 20° (Fig. 3). In this position, the least amount of tension is placed on the ligaments, muscles, and tendons of the hand [23]. Maintaining this neutral wrist position in unloaded and loaded situations to obtain a functional and stable joint position and adequate kinesthesia will be emphasized. The participants will also be equipped with a stable wrist orthosis with the instruction to wear it, particularly during pain-provoking activities, but also at night-time if needed (Table 1).

**Table 1:** Structured education regarding wrist anatomy, pathophysiology, and self-management strategies

Topics	Objectives
**Anatomy of the wrist**	Know the basic anatomy and biomechanics of the wrist
**Pathophysiology**	Know the pathophysiology of wrist osteoarthritis and its symptoms and risk factors
**Joint protective self-management strategies**	Knowledge about: - The awareness of keeping the wrist in a stable neutral position in activities of daily living - Avoiding a monotonous load on the wrist for a long time - The awareness of compensatory movements and pain-provoking activities - Taking regular and frequent breaks when needed - Protecting the wrist with a wrist orthosis (Prisma stabil plus™ or Wrist Lacer II™) - Using ergonomic tools when needed
**Management of pain and fatigue by exercises**	Knowledge about: - The benefits and the purpose of the allocated exercise program - The importance of performing the exercises in a pain-free manner and with good quality of movement - The importance of adhering to the allocated program

### Adherence

Before randomization and during all contacts with the treating PT, the participants will be informed about the importance of adhering to the treatment program. Participants will be followed up by the treating PT at the clinic at 2, 6, and 12 weeks after baseline, and by phone at 4 and 8 weeks after baseline, to ensure adherence to the program and that the regimen and exercises are carried out correctly. At the appointments, the participants will show and repeat their exercises, and adjustments and corrections will be made when needed. They will also be asked about whether they are able to adhere to the program or if they find the exercise regimen challenging. All contact with the participants will be documented in their medical journals.

### Exercise therapy for the intervention group

The exercise therapy program for the intervention group has been designed by the first author (SL) having applied findings from previous studies on wrist stability and proprioception [22, 24–26]. Focus is on functional re-learning and strengthening of the musculoskeletal system with the aim to create a stable wrist that can be used in a pain-free manner in daily activities [22]. A clear understanding of the program and good motivation are essential. The emphasis will therefore lie on a thorough perception of the rationale and goal of the exercise therapy program.

**Fig. 4:**
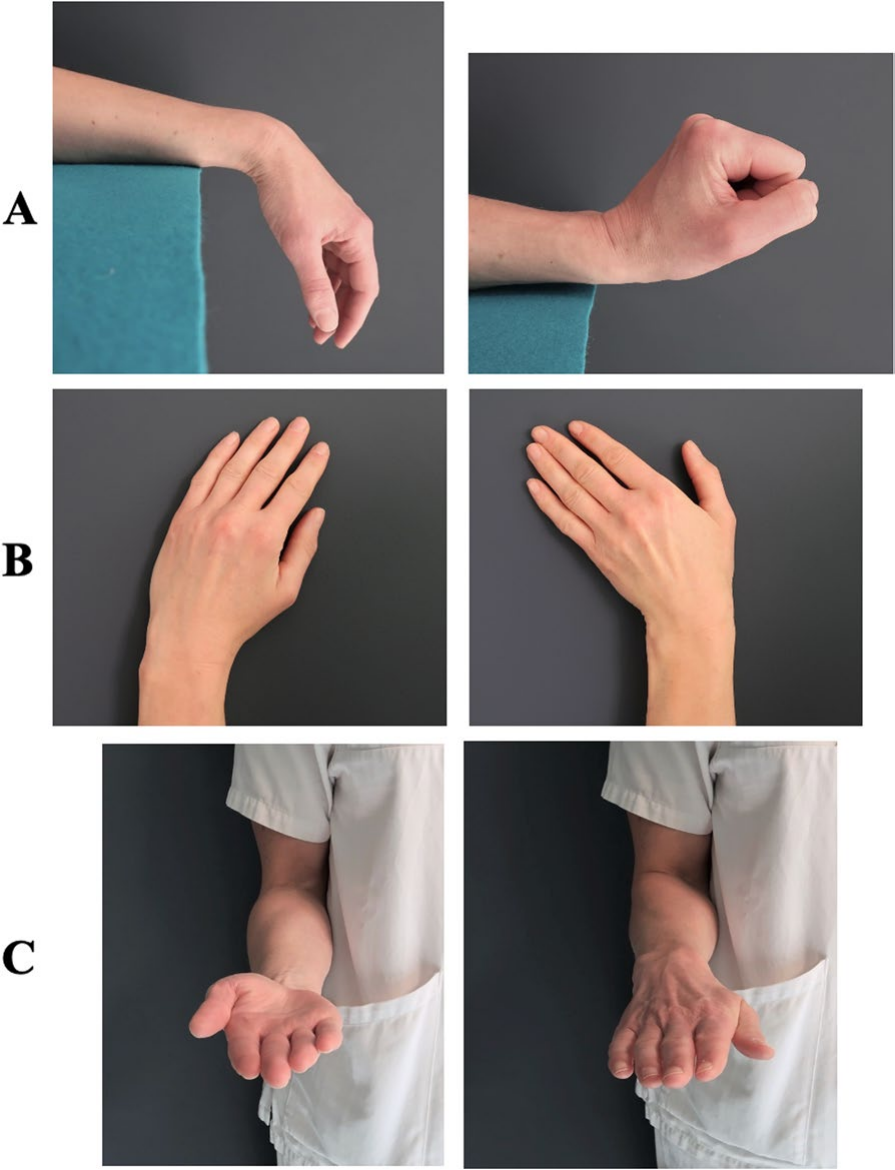
The range of wrist motion training program performed by both groups. **A** Flexion and extension. **B** Radial and ulnar deviation. **C** Supination and pronation. All exercises will be completed with 10 × 2 repetitions for the control group and 10 repetitions for the intervention group. Both groups will perform the exercises in a pain-free manner two times per day for 12 weeks

**Fig. 5:**
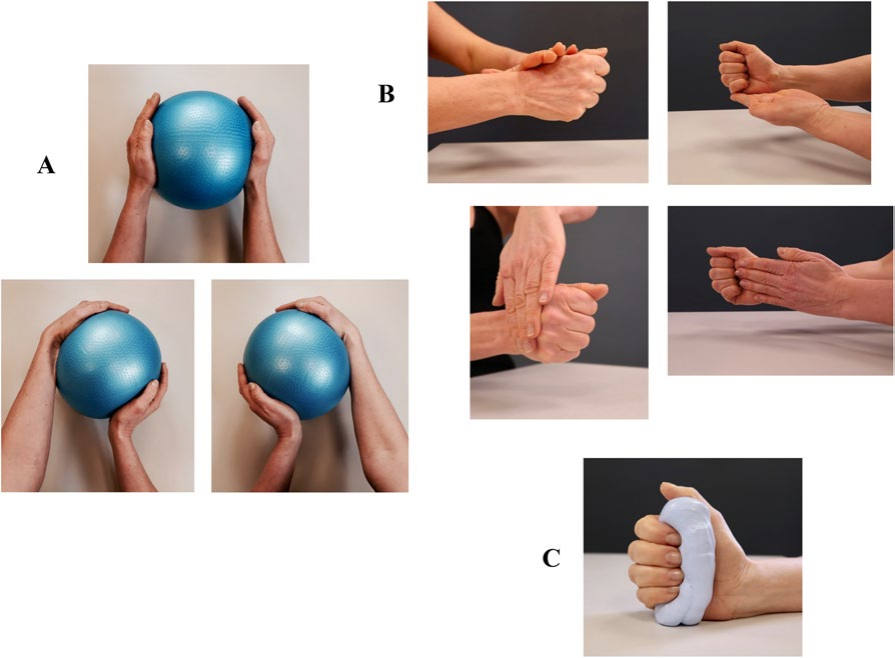
The exercise therapy program performed by the intervention group. **A** Coordination and co-activation exercise. This is a closed-chain isometric and active range of wrist motion exercise with a ball, training co-activation, and coordination. **B** Isometric exercise. The participants will apply manual isometric resistance for the long extrinsic muscles of the wrist, while at the same time maintaining a stable and neutral position. **C** Strength exercise. The participants will squeeze a silicon putty dough while maintaining the wrist in a neutral position. All exercises (**A**–**C**), including the range of wrist motion exercises (Fig. 4), will be performed in a pain-free manner by the intervention group two times per day for 12 weeks

The program consists of two parts. The first part contains unloaded active ROM exercises for the wrist in flexion/extension, radial-/ulnar deviation, and pronation/supination (Table 2 and Fig. 4). The second part of the program consists of neuromuscular exercises (described below in A to C) that focus on coordination, wrist stability, and strength (Table 3 and Fig. 5).

A. *Coordination and co-activation exercise*. The first exercise is training the coordination and co-activation of the long extrinsic muscles acting as active stabilizers of the wrist [the extensor carpi radialis longus and brevis muscles (ECRL/ECRB), the extensor carpi ulnaris muscle (ECU), the flexor carpi radialis muscle (FCR), and the flexor carpi ulnaris muscle (FCU)] [24]. Co-activation exercises demand the use of eccentric, concentric, and isometric exercises and will be performed as closed-chain isometric and active exercises with both hands on a ball [24].
B. *Isometric exercise*. The participants will apply manual isometric resistance for the extrinsic muscles of the wrist (ECRL/ECRB, ECU, FCR, FCU) while at the same time maintaining a neutral position. Isometric exercises are user-friendly, build muscle strength quickly, and appear to have a key role in functional wrist motor re-learning [22, 24].
C. *Strength exercise*. Grip strength and strength of the extrinsic muscles around the wrist will be trained. In this exercise, the participants will squeeze a silicon putty dough while at the same time maintaining the wrist in a neutral position.

**Table 2:** Details of the range of wrist motion program including the exercises, performance, and repetitions

**Range of wrist motion exercises**	Active range of wrist motion in: A. Flexion and extension B. Radial and ulnar deviation C. Supination and pronation
**Performance and repetitions**	The participants will perform the exercises slowly and with good quality of movement. They will hold in a pain-free outer position for about 3–5 s and repeat the exercises: **Control group**: 10 × 2 repetitions **Intervention group**: 10 repetitions

**Table 3:** Details of the exercise therapy program including the exercises, performance, and repetitions

Exercises	Performance and repetitions
**A.** **Coordination and co-activation exercise**	Closed-chain isometric and active range of wrist motion exercise with a ball, training coordination, and co-activation Participants will be taught to sit with the ball placed on the table. They will be instructed to gently press both hands against the ball, holding for 3–5 s. After that, they will gently press their palms against the ball while slowly turning the ball to the right and left in a pain-free manner They will perform the exercise with 10 repetitions
**B.** **Isometric exercise**	Isometric exercise, where the participants will apply moderate resistance to their affected wrist with their other hand in the opposite direction of the wrist movement They will hold for 3–5 s and perform the exercise with 10 × 2 repetitions
**C.** **Strength exercise**	Silicone putty dough exercise for grip strength and strength of the extrinsic muscles around the wrist The participants will be taught to hold their wrist in a stable neutral position at the whole time during the exercise They will hold for 3–5 s and perform the exercise with 10 × 2 repetitions

### Range of wrist motion exercises for the control group

The training program for the control group will, just as the intervention group, consist of home base exercises twice a day for 12 weeks. However, the training program will only include the above-mentioned ROM exercises (Table 2 and Fig. 4).

## Outcome measures

Outcome measures with good psychometric properties will be used covering both physical and patient-reported measures. Valid and reliable Swedish versions of the outcome measures will be used. Missing item responses for the patient-reported outcome measures will be handled in accordance with each scale’s specific recommendations. If the number of missing items makes calculating the score impossible, the lost score will not be replaced. Outcomes will be assessed at baseline and at 3, 6, and 12 months post-inclusion (Fig. 2).

### Primary outcome measure

#### Patient-Rated Wrist Evaluation (PRWE)

PRWE is a wrist specific patient-rated outcome measure (PROM) originally developed for the assessment of perceived disability after a distal radius fracture [18]. However, strong psychometric properties of PRWE, such as excellent test–retest reliability and high construct validity, have been found in patients with wrist OA [27]. The questionnaire includes 15 questions, divided into two subscales assessing pain (5 items) and function (10 items, 6 concerning specific tasks and 4 the ability to perform daily activities) over the past week [18]. The questions are scored on a 10-point ordered categorical scale, ranging from no pain or no difficulty (0 points) to worst pain or unable to do (10 points). The total score of the subscales pain (sum of 5 items) and function (sum of 10 items divided by 2) ranges from 0 to 50. The maximum total score of PRWE is 100 and represents the worst disability, whereas 0 represents no disability. In this RCT, the Swedish version of PRWE, which is a responsive, valid, and reliable patient-rated outcome measure, will be used [28].

### Secondary outcome measures

#### Hand dynamometer (grip strength)

The isometric grip strength will be measured on both hands using the Jamar hydraulic hand dynamometer according to standardized instructions [29, 30]. The same hand dynamometer will be used for all measurements (TEC, Clifton, NJ, USA). Three trials for each hand will be recorded, and the mean value, recorded in kilograms (kg), for each hand will be calculated.

#### Goniometer (range of wrist motion)

Range of wrist motion (flexion, extension, radial deviation, ulnar deviation, pronation, and supination) of the affected wrist will be measured with a goniometer according to standardized instructions [30, 31]. We expect the intra-rater reliability of the goniometry measurements to be high, since all measurement will we performed by the same experienced PT [32].

#### Numerical Pain Rating Scale (NPRS)

The NPRS is a numeric 11-point pain rating box scale with numerical descriptors on the box, ranging from 0 representing one pain extreme (no pain) to 10 representing the other pain extreme (worst pain imaginable) [33]. Participants select a value that is most in line with the intensity of pain they have perceived in the affected wrist over the last week. Three measures of pain will be rated in this trial: (1) pain at rest, (2) pain on motion without load, and (3) pain on load. The NPRS have been found to be valid and reliable when measuring pain outcome in patients with wrist OA [27].

#### Disabilities of the Arm, Shoulder, and Hand (DASH)

The DASH measures self-reported upper extremity physical function and symptoms taking the whole upper extremity into account, irrespective of which hand or if both hands are used [34]. Excellent test–retest reliability and moderate to high construct validity have been found for DASH in patients with wrist OA [27]. The main part of the DASH is a 30-item disability/symptom scale concerning the patient’s health status during the preceding week. The items ask about the degree of difficulty in performing different physical activities because of arm, shoulder, or hand problems (21 items) and the severity of each of the symptoms of pain, activity-related pain, tingling, weakness, and stiffness (5 items), as well as the problem’s impact on social activities, work, sleep, and self-image (4 items). Each item has five response options. The scores for all items are then used to calculate a scale score ranging from 0 (no disability) to 100 (most severe disability). The validated Swedish version of DASH will be used in this RCT [35].

#### Generalized Self-Efficacy Scale (GSES)

A significant determinant of health behavior is self-efficacy, or the individual’s belief that he or she can successfully complete a goal or behavior to achieve a desired outcome. The GSES was developed to assess the strength of a person’s belief in his or her ability to respond to novel or difficult situations and to deal with any associated obstacles or setbacks [36]. The GSES is a ten-item scale, where each item ranges from 1 (“not at all true”) to 4 (“exactly true”). Scores are summed across the ten-items to give a total score, with a possible range of 10–40. Higher scores indicate greater confidence in generalized self-efficacy. The validated Swedish version of the GSES will be used [37].

#### Global Rating of Change (GROC)

The GROC score measure self-perceived change in health status over time and have become widely used in both research settings and clinical practice for determination of the clinically important change and measurement of outcome [38]. The GROC score involves a single question that asks the participant to rate their change with respect to a particular condition from the time they began treatment until the time they answered the question. The rating is based on a 11- point self-report Likert scale (from − 5 to 5), where a “ − 5” indicates “a very great deal worse,” “0” indicates “about the same,” and “ + 5” indicates “a very great deal better”. At the follow-ups, the participants will be asked to “rate the overall change” and respond to the question: “Regarding your wrist problems, how would you describe your wrist now compared to before the training period?” The GROC scale has the advantages of clinical relevance, adequate reproducibility, and sensitivity to change and is intuitively easy to understand by the patient [38].

#### Conversion to surgery

Conversion to surgical interventions will be based on the participants’ symptoms and wishes and the surgeon’s recommendation. This decision will be made during a face-to-face appointment with the participant and the hand surgeon at 3–6 months following randomization. The hand surgeon will base their recommendation on surgery given the participants presentation and symptom severity at the appointment. Participants that are ambivalent about surgery will be offered another follow-up visit or telephone contact with the hand surgeon at a later stage. The percentage of participants requiring surgery in both groups will be compared. Comparison of conversion to surgery has been used in previous clinical trials to determine the success of non-surgical management [39].

## Statistical analysis

### Assessments of efficacy

Primary endpoint:

1. PRWE at 3 months

Secondary endpoints:

1. PRWE at 6 and 12 months
2. DASH at 3, 6, and 12 months
3. NPRS pain at 3, 6, and 12 months
4. GSES at 3, 6, and 12 months
5. Grip strength and wrist ROM at 3, 6, and 12 months
6. GROC at 3, 6, and 12 months
7. Conversion to surgery at 6 and 12 months

### Data analysis

Appropriate descriptive statistics for all outcome measures and demographic characteristics for the intervention and control groups will be reported for baseline and 3, 6, and 12 months. For continuous variables, mean values and standard deviations (SD) will be calculated. If the continuous data is not normally distributed, median values and interquartile range (IQR) will instead be calculated. For categorical variables, median and IQR will be calculated, and for binary variables, proportions and percentages will be conducted. Baseline data from possible dropouts will be described and compared to the included participants. The primary results will be interpreted based on the intention-to-treat principle. In case of crossovers, sensitivity analysis comparing the results of intention-to-treat and per-protocol analyses will be performed and reported. To analyze differences between the groups at baseline and at 3, 6, and 12 months, the Mann–Whitney test (for ordinal data) or independent sample *t*-test (for continuous data) will be used depending on normal distribution, examined by the Shapiro–Wilk test. The Wilcoxon signed-rank test or paired *t*-test will be used to analyze within-group differences. Conversion to surgery will be analyzed by a chi-square test or a Fisher’s exact test. Missing data will be managed according to the algorithm described by Jakobsen et al., using multiple imputation if complete case analyses are not supported [40]. The level of statistical significance will be set at *p* < 0.05. All calculations will be performed using IBM SPSS Statistics version 29 (IBM Corporation, Armonk, NY, USA).

### Withdrawal and safety

The participants will be able to withdraw from the trial at any time, without giving an explanation and without any negative consequences for their care and rehabilitation in the future according to the ethical permission. Based on international and national guidelines recommending education and exercise as core treatment of OA [41], our judgment is that there is low risk of adverse events in this RCT.

### Retention plan

To promote participant retention, written educational materials to ensure that the participants fully understand the purpose, procedures, risks, and benefits of the trial will be developed. The participants will also be given trial related materials, such as a calendar, to help them track their participation, adhere to procedures, and maintain engagement. During the trial process, regular visits and communication will be maintained to ensure adherence and non-retention. If the participants cannot come to the clinic for a follow-up, they will be offered an online meeting instead. Participants, who do not wish to attend physical examinations, will be asked to fill in the questionnaires at home and send them to the research team. Flexible and convenient scheduling options for visits will be offered. The visits to the treating PT will be free of charge during the trial and the participants will also be offered appropriate reimbursement for any travel expenses. User-friendly data collection assessments to minimize errors will be used and regular monitoring of the data collection to identify missing data will be implemented.

### Data management

Data from all assessments will be decoded and stored in binders and in a secure database. The decoding key will be locked in a safe and the database will be protected by a password to which only researchers responsible for the trial have access. After completion of the trial, all files will be saved for at least 10 years according to national rules.

## Discussion

An effective self-management treatment program, including structured education and therapeutic exercises, has not yet been introduced for individuals with wrist OA. Although there is no cure for OA, patients may benefit from self-management treatment options that enables them to manage symptoms and optimize quality of life [42]. There is strong evidence advocating that all patients with OA should be offered adequate education and exercises and that surgical interventions only should be considered when non-surgical treatments have failed [41]. In addition, worldwide, waiting times are long before consultation with a specialist or elective surgery is received, which emphasizes the need for physiotherapist-led interventions and new conservative treatment options [43]. Referral of patients with wrist OA to education and self-management programs is therefore an attractive first-line treatment option with the intention to inform patients about the disease and provide them with tools to facilitate everyday life and with the aim to postpone, or even eliminate, the need for surgical interventions [39]. Thus, evaluation of the present self-managed exercise therapy program may be beneficial for the individual who suffers from wrist OA as well as for the healthcare system.

Several international and national organizations recommend structured education and exercises as first-line treatment for OA. The European League Against Rheumatism (EULAR) recommends OA education and exercises for hand, knee, and hip OA [17, 44]. The Osteoarthritis Research Society International (OARSI) recommends education and exercise programs as core treatments for knee, hip, and polyarticular OA [13]. Furthermore, several evidence-based self-management programs have been developed for hip and knee OA and are implemented and used as first-line treatments with good results [16, 45]. Cochrane reviews have found high quality evidence that therapeutic exercises can reduce pain and improve function in knee and hip OA [14, 15]. For hand OA, however, the evidence is low owing to lack of blinding of participants, the small number of included studies, and inclusion of few individuals in the analyses [46]. In these studies, hand OA refers to the thumb carpometacarpal (CMC) joint and finger joint OA. For wrist OA, structured education, and exercise therapy as first-line treatment has not yet been studied.

A structured education and neuromuscular exercise therapy program is one type of conservative management for hip and knee OA [42]. Our exercise therapy program is designed based on the principles behind neuromuscular training that focus on improving the quality and effectiveness of movements [12, 22]. The rationale behind our choice of neuromuscular therapeutic exercises is that patients with OA may have impaired sensorimotor function in terms of sensory deficiency, altered muscle activation patterns, and reduced functional performance [9, 47]. Therefore, it seems evident that training programs should address several aspects of the sensorimotor system to improve function and alleviate symptoms.

The combination of structured education and neuromuscular exercise therapy has shown short- and long-term improvements in pain, physical function, function in ADL, and quality of life in individuals with knee and hip OA [48–51]. Skou et al. evaluated the efficacy of a 12-week non-surgical treatment program for patients with knee OA not eligible for total knee replacement in a RCT. They found that participants in a structured education and neuromuscular exercise group experienced significant improvements regarding pain, function, and quality of life after 1 year compared to the control group receiving usual care consisting of two leaflets with information and advice on knee OA and recommended treatments [49]. In a large study, 418 patients with chronic knee pain/knee OA were randomized to either usual care or the Enabling Self-Management and Coping of Arthritic Knee Pain Through Exercise (ESCAPE) program. Significant improvements in pain and physical function were found in the ESCAPE group at 6 weeks post-intervention and the improvement sustained at a 30-month follow-up [48]. Da Silva et al. demonstrated, in an RCT, significant improvements in pain, physical function, ADL, and quality of life at the 8-week follow-up in the structured education and neuromuscular exercise group compared to patient education for patients with knee OA [50]. A 6-year follow-up RCT in patients with hip OA by Svege et al. found significant improvement in self-reported physical function for participants in the structured education and neuromuscular exercise group compared to the control group that only received patient education. The study also found that exercise therapy in addition to patient education can reduce the need for total hip replacement by 44% in patients with hip OA [51]. The wrist, with its complex anatomy and biomechanics, cannot be fully compared to weight-bearing joints such as the hip and knee, which emphasizes the need to evaluate if a self-management program, including patient education and neuromuscular exercises, can be beneficial also for individuals with wrist OA.

A review by Hagert from 2010 [24] highlighted the theoretical importance of sensorimotor control of the wrist and its function, thus setting a new standard for wrist rehabilitation. Clinical reviews by Valdes et al. [52], Karagiannopoulos and Michlovitz’s [26], and Lotters et al. [25] provided further support for including exercises of sensorimotor control in the rehabilitation of the wrist. In addition, there are a small number of single case reports [53, 54] and cohort studies [22, 55, 56], indicating clinical benefits of an exercise therapy program with a neuromuscular approach following various wrist injuries. The exercise therapy in our RCT is based on theoretical assumptions and outcomes from the above-mentioned cohort and case studies.

A potential limitation to our RCT is the fact that adherence may be more challenging for the participants in the intervention group due to the larger training intensity. To promote adherence, both treatment programs will be delivered and supervised by an experienced PT who will encourage, answer questions, and initiate individual adjustments when needed. Moreover, there may be a risk of self-selection bias; thus, individuals that accept to join the trial may differ in terms of motivation to training compared to those who chose not to participate.

Since the clinical importance of wrist rehabilitation remains in its infancy, we want to evaluate the concept of structured education and a self-management exercise therapy program as treatment for individuals with wrist OA.

## Conclusion

We have designed a self-managed exercise therapy program for individuals with wrist OA that we will evaluate in a single-blinded RCT with two treatment arms. The knowledge gained will comprehend the effectiveness of this non-surgical treatment and, depending on the outcome, may redefine the current treatment strategies. If this self-management program proves to be effective, in terms of decreased pain and improved patient-reported function, it may be implemented in treatment protocols for individuals with wrist OA.

## Trial status

This protocol is version 3.0 (dated August 18, 2023). The recruitment of participants started 3 October 2019 and is estimated to be completed in June 2023. No trial amendments have been made since the enrollment of the first participants. Any substantive amendments that may impact the conduct of the trial or the ethical rigor will require a formal written modification to the protocol and an approval by the Swedish Ethical Review Authority.

## Dissemination policy

The findings of this trial will be communicated to the participants and disseminated through peer-reviewed publications in scientific journals and conference presentations. It is also expected to, through presentations, generate a linkage with specialized hospitals and therapists working in this field.

### Supplementary Information

**Additional file 1.** SPIRIT Checklist for Trials.

**Additional file 2.** Consent form.

## Data Availability

The datasets that will be generated and/or analyzed during the current trial will not be publicly available. Public access to data is restricted by the Swedish government (Public Access to Information and Secrecy Act; https://www.government.se/information-material/2009/09/public-access-to-information-and-secrecy-act/). Data may be available for researchers upon special review and includes approval of the research project by both an Ethics Committee at national level, governmental data safety committees, and the regional committee at the health care sector in Region Skåne, Sweden.

## Abbreviations

ADL: Activities of daily living
CMC: Carpometacarpal
CT: Computer tomography
DASH: Disabilities of the Arm, Shoulder, and Hand
ECRB: Extensor carpi radialis brevis
ECRL: Extensor carpi radialis longus
ECU: Extensor carpi ulnaris
ESCAPE: The Enabling Self-Management and Coping of Arthritic Knee Pain Through Exercise
EULAR: The European League Against Rheumatism
FCR: Flexor carpi radialis
FCU: Flexor carpi ulnaris
GROC: Global Rating of Change
GSES: Generalized Self-Efficacy Scale
MCID: Minimal clinically important difference
NPRS: Numeric Pain Rating scale
NSAID: Non-steroidal anti-inflammatory drug
OA: Osteoarthritis
OARSI: The Osteoarthritis Research Society International
OT: Occupational therapist
PROM: Patient-rated outcome measure
PRWE: Patient-Rated Wrist Evaluation
PT: Physiotherapist
RCT: Randomized controlled trial
ROM: Range of motion
SD: Standard deviation
SLAC: Scapholunate advanced collapse
SNAC: Scaphoid non-union advanced collapse
SPIRIT: The Standard Protocol Items: Recommendations for Interventional Trials
