# Supplementary material for: A self-managed exercise therapy program for wrist osteoarthritis: study protocol for a randomized controlled trial
Source: Trials. 2023 Oct 2;24:628. doi: 10.1186/s13063-023-07668-4 (PMC10546651; doi:10.1186/s13063-023-07668-4)
Supplement: Supplementary file 1 — Additional file 1. SPIRIT Checklist for Trials. [file 13063_2023_7668_MOESM1_ESM.pdf]

SPIRIT Checklist for *Trials*

Complete this checklist by entering the page and line numbers where each of the items listed below can be found in your manuscript.

Your manuscript may not currently address all the items on the checklist. Please modify your text to include the missing information. If you are certain that an item does not apply, please state "n/a" and provide a short explanation. **Leaving an item blank or stating "n/a" without an explanation will lead to your manuscript being returned before review.**

Upload your completed checklist as an additional file when you submit to *Trials*. You must reference this additional file in the main text of your protocol submission. The completed SPIRIT figure must be included within the main body of the protocol text and can be downloaded here: http://www.spirit-statement.org/schedule-of-enrolment-interventions-and-assessments/

In your methods section, please state that you used the SPIRIT reporting guidelines, and cite them as:

Chan A-W, Tetzlaff JM, Gøtzsche PC, Altman DG, Mann H, Berlin J, Dickersin K, Hróbjartsson A, Schulz KF, Parulekar WR, Krleža-Jerić K, Laupacis A, Moher D. SPIRIT 2013 Explanation and Elaboration: Guidance for protocols of clinical trials. BMJ. 2013;346:e7586

		Reporting Item	Page and Line Number	Reason if not applicable
**Administrative information**				
Title	#1	Descriptive title identifying the study design, population, interventions, and, if applicable, trial acronym	1 (1-2)	
Trial registration	#2a	Trial identifier and registry name. If not yet registered, name of intended registry	5 (80-81)	
Trial registration: data set	#2b	All items from the World Health Organization Trial Registration Data Set	5 (80-81)	See item 2a and registration in ClinicalTrials.gov, registry number NCT05367817.
Protocol version	#3	Date and version identifier	27 (549)	

Funding	#4	Sources and types of financial, material, and other support	2 (40-45)	
Roles and responsibilities: contributorship	#5a	Names, affiliations, and roles of protocol contributors	1-3 (4-18, 46-49)	
Roles and responsibilities: sponsor contact information	#5b	Name and contact information for the trial sponsor	2 (40-45)	This is an investigator initiated clinical trial. Therefore, the funders played no part in study design, data collection, management, analysis, interpretation of data, writing of the report, and the decision to submit the report for publication.
Roles and responsibilities: sponsor and funder	#5c	Role of study sponsor and funders, if any, in study design; collection, management, analysis, and interpretation of data; writing of the report; and the decision to submit the report for publication, including whether they will have ultimate authority over any of these activities	n/a	This is not applicable since this is an investigator initiated clinical trial (see item 5b).
Roles and responsibilities: committees	#5d	Composition, roles, and responsibilities of the coordinating centre, steering committee, endpoint adjudication committee, data management team, and other individuals or groups overseeing the trial, if applicable (see Item 21a for data monitoring committee)	n/a	No committees or coordination centers are involved in the study.

**Introduction**				
Background and rationale	#6a	Description of research question and justification for undertaking the trial, including summary of relevant studies (published and unpublished) examining benefits and harms for each intervention	6-7 (102-129)	
Background and rationale: choice of comparators	#6b	Explanation for choice of comparators	6-7 (102-129)	
Objectives	#7	Specific objectives or hypotheses	7 (124-129)	
Trial design	#8	Description of trial design including type of trial (eg, parallel group, crossover, factorial, single group), allocation ratio, and framework (eg, superiority, equivalence, non-inferiority, exploratory)	8 (131-138)	
**Methods: Participants, interventions, and outcomes**				
Study setting	#9	Description of study settings (eg, community clinic, academic hospital) and list of countries where data will be collected. Reference to where list of study sites can be obtained	9 (165-169)	
Eligibility criteria	#10	Inclusion and exclusion criteria for participants. If applicable, eligibility criteria for study centres and individuals who will perform the interventions (eg, surgeons, psychotherapists)	9 (155-164)	

Interventions: description	#11a	Interventions for each group with sufficient detail to allow replication, including how and when they will be administered	12-17 (241-316)	
Interventions: modifications	#11b	Criteria for discontinuing or modifying allocated interventions for a given trial participant (eg, drug dose change in response to harms, participant request, or improving / worsening disease)	11 (211-213), 23 (436-440)	
Interventions: adherance	#11c	Strategies to improve adherence to intervention protocols, and any procedures for monitoring adherence (eg, drug tablet return; laboratory tests)	14-15 (271-280)	
Interventions: concomitant care	#11d	Relevant concomitant care and interventions that are permitted or prohibited during the trial	13 (252-2258)	
Outcomes	#12	Primary, secondary, and other outcomes, including the specific measurement variable (eg, systolic blood pressure), analysis metric (eg, change from baseline, final value, time to event), method of aggregation (eg, median, proportion), and time point for each outcome. Explanation of the clinical relevance of chosen efficacy and harm outcomes is strongly recommended	17-21(317-403), 21-22 (405-415)	
Participant timeline	#13	Time schedule of enrolment, interventions (including any run-ins and washouts), assessments, and visits for participants. A schematic diagram is highly recommended (see Figure)	8 (137-138, Figure 1 and 2), 12-13 (242-251), 14 (272-276), 18 (323)	

Sample size	#14	Estimated number of participants needed to achieve study objectives and how it was determined, including clinical and statistical assumptions supporting any sample size calculations	8 (139-152)	
Recruitment	#15	Strategies for achieving adequate participant enrolment to reach target sample size	9-10 (170-180)	
**Methods: Assignment of interventions (for controlled trials)**				
Allocation: sequence generation	#16a	Method of generating the allocation sequence (eg, computer-generated random numbers), and list of any factors for stratification. To reduce predictability of a random sequence, details of any planned restriction (eg, blocking) should be provided in a separate document that is unavailable to those who enrol participants or assign interventions	10 (186-198)	
Allocation concealment mechanism	#16b	Mechanism of implementing the allocation sequence (eg, central telephone; sequentially numbered, opaque, sealed envelopes), describing any steps to conceal the sequence until interventions are assigned	10 (189-195)	
Allocation: implementation	#16c	Who will generate the allocation sequence, who will enrol participants, and who will assign participants to interventions	10 (187-197)	
Blinding (masking)	#17a	Who will be blinded after assignment to interventions (eg, trial participants, care	11 (199-211)	

		providers, outcome assessors, data analysts), and how		
Blinding (masking): emergency unblinding	#17b	If blinded, circumstances under which unblinding is permissible, and procedure for revealing a participant's allocated intervention during the trial	11 (211-215)	
**Methods: Data collection, management, and analysis**				
Data collection plan	#18a	Plans for assessment and collection of outcome, baseline, and other trial data, including any related processes to promote data quality (eg, duplicate measurements, training of assessors) and a description of study instruments (eg, questionnaires, laboratory tests) along with their reliability and validity, if known. Reference to where data collection forms can be found, if not in the protocol	17-22 (317-415), Figure 1 and 2.	
Data collection plan: retention	#18b	Plans to promote participant retention and complete follow-up, including list of any outcome data to be collected for participants who discontinue or deviate from intervention protocols	23 (441-454)	
Data management	#19	Plans for data entry, coding, security, and storage, including any related processes to promote data quality (eg, double data entry; range checks for data values). Reference to where details of data management procedures can be found, if not in the protocol	23 (455-459)	

Statistics: outcomes	#20a	Statistical methods for analysing primary and secondary outcomes. Reference to where other details of the statistical analysis plan can be found, if not in the protocol	22 (416-434)	
Statistics: additional analyses	#20b	Methods for any additional analyses (eg, subgroup and adjusted analyses)	n/a	We are not planning additional, subgroup or adjusted analyses
Statistics: analysis population and missing data	#20c	Definition of analysis population relating to protocol non-adherence (eg, as randomised analysis), and any statistical methods to handle missing data (eg, multiple imputation)	22 (422-424), 22 (431-432), 17 (320-321)	
**Methods: Monitoring**				
Data monitoring: formal committee	#21a	Composition of data monitoring committee (DMC); summary of its role and reporting structure; statement of whether it is independent from the sponsor and competing interests; and reference to where further details about its charter can be found, if not in the protocol. Alternatively, an explanation of why a DMC is not needed	n/a	A DMC is considered not necessary since the interventions (two different rehabilitation programs for wrist OA) implies minimal risk for the study participants.
Data monitoring: interim analysis	#21b	Description of any interim analyses and stopping guidelines, including who will have access to these interim results and make the final decision to terminate the trial	n/a	An interim analysis will not be part of the trial design since the interventions implies minimal risk for the study participants.
Harms	#22	Plans for collecting, assessing, reporting, and managing solicited and spontaneously reported	23 (436-440)	

		adverse events and other unintended effects of trial interventions or trial conduct		
Auditing	#23	Frequency and procedures for auditing trial conduct, if any, and whether the process will be independent from investigators and the sponsor	n/a	No auditing is planned.
**Ethics and dissemination**				
Research ethics approval	#24	Plans for seeking research ethics committee / institutional review board (REC / IRB) approval	2 (25-29), 10 (181-185)	
Protocol amendments	#25	Plans for communicating important protocol modifications (eg, changes to eligibility criteria, outcomes, analyses) to relevant parties (eg, investigators, REC / IRBs, trial participants, trial registries, journals, regulators)	27 (550-553)	
Consent or assent	#26a	Who will obtain informed consent or assent from potential trial participants or authorised surrogates, and how (see Item 32)	2 (27-29), 10 (183-183)	
Consent or assent: ancillary studies	#26b	Additional consent provisions for collection and use of participant data and biological specimens in ancillary studies, if applicable	n/a	There will be no collection of additional participant data or biological specimens to ancillary studies
Confidentiality	#27	How personal information about potential and enrolled participants will be collected, shared, and maintained in order to protect confidentiality before, during, and after the trial	2 (31-38), 23 (45-459)	
Declaration of interests	#28	Financial and other competing interests for principal investigators for the overall trial and each study site	2 (39)	

Data access	#29	Statement of who will have access to the final trial dataset, and disclosure of contractual agreements that limit such access for investigators	2 (31-38)	
Ancillary and post trial care	#30	Provisions, if any, for ancillary and post-trial care, and for compensation to those who suffer harm from trial participation	13 (249-251)	
Dissemination policy: trial results	#31a	Plans for investigators and sponsor to communicate trial results to participants, healthcare professionals, the public, and other relevant groups (eg, via publication, reporting in results databases, or other data sharing arrangements), including any publication restrictions	28 (554-558)	
Dissemination policy: authorship	#31b	Authorship eligibility guidelines and any intended use of professional writers	3 (46-49)	
Dissemination policy: reproducible research	#31c	Plans, if any, for granting public access to the full protocol, participant-level dataset, and statistical code	2 (31-38)	
**Appendices**				
Informed consent materials	#32	Model consent form and other related documentation given to participants and authorised surrogates	10 (182-183, Appendix 1)	
Biological specimens	#33	Plans for collection, laboratory evaluation, and storage of biological specimens for genetic or	n/a	Biological specimens will not be collected.

		molecular analysis in the current trial and for future use in ancillary studies, if applicable		

It is strongly recommended that this checklist be read in conjunction with the SPIRIT 2013 Explanation & Elaboration for important clarification on the items. Amendments to the protocol should be tracked and dated. The SPIRIT checklist is copyrighted by the SPIRIT Group under the Creative Commons "Attribution-NonCommercial-NoDerivs 3.0 Unported" license. This checklist can be completed online using https://www.goodreports.org/, a tool made by the EQUATOR Network in collaboration with Penelope.ai
