# Supplementary material for: A self-managed exercise therapy program for wrist osteoarthritis: study protocol for a randomized controlled trial
Source: Trials. 2023 Oct 2;24:628. doi: 10.1186/s13063-023-07668-4 (PMC10546651; doi:10.1186/s13063-023-07668-4)
Supplement: Supplementary file 2 — Additional file 2. Consent form. [file 13063_2023_7668_MOESM2_ESM.pdf]

Region Skåne, Skåne University hospital Malmö, Sara Larsson, cert. Physioterapist, Handsurgery Rehab, SE-205 02 Malmö, Sweden. +46 40 33 6706

Patient information – investigation of how neuromuscular training affects wrist function in people with wrist osteoarthritis

You are hereby asked if you would like to participate in a research study investigating whether neuromuscular training can improve wrist function in people with wrist osteoarthritis.

Before you decide, please read through this information, and ask if there's anything you don't understand or if there's anything you're wondering about. If you choose to participate in the study, you will be asked to sign a consent form.

Background

Neuromuscular training includes exercises that combine mobility, strength, coordination, and balance in order to improve stability and strength in and around a joint. There is a lot of research showing that this type of exercise improves function and reduces pain in patients with knee and hip osteoarthritis, but there are no previous studies that have examined whether neuromuscular training can improve function and reduce pain in people with wrist osteoarthritis.

Aim

The aim of the study is to investigate the effect of neuromuscular training in people with wrist osteoarthritis.

How does the study work?

If you choose to participate in the study, you will be randomly assigned to one of two different groups. Group 1 will receive common traditional treatment of wrist osteoarthritis, including a wrist orthosis for pain relief, range of wrist motion training within the pain threshold and ergonomic advice. Group 2 will, in addition to the traditional treatment, also receive neuromuscular training of the wrist that includes muscle strengthening exercises.

In order to assess the degree of osteoarthritis, a computer tomography (CT) scan, on your wrist will be performed. After this, a visit will be booked to the responsible physiotherapist at the Hand Surgery Clinic's rehabilitation department, Skåne University Hospital, Malmö.
In connection with this visit, you will receive instructions on how the treatment and training should be carried out. Thereafter, follow-up with the same physiotherapist will take place about 10-14 days and 6 weeks after the first visit. These visits take about 30 minutes. A telephone follow-up will take place after 4 and 8 weeks.

The efficacy of the training will be evaluated 3, 6 and 12 months after treatment start in both groups.

The evaluation takes about 60 minutes and consists of 2 parts:

1. 1. Clinical examination. A physiotherapist measures wrist mobility, strength, and grip force in both hands. You get to estimate how much pain you have during rest, at movement, and under load on a 10-point scale.

2. Questionnaires. You will fill in three questionnaires about how you experience the function of your hand and wrist and how you experience your confidence in your ability to cope with different situations.

Possible discomforts and risks

Traditional training and also neuromuscular training do not bring any discomfort or risks. The evaluations made during the follow-ups do not cause any discomfort or risks.

Possible benefits

Exercise improves function and reduces pain in patients with osteoarthritis of the knee and hip. It is therefore likely that exercise can also improve function and reduce pain in patients with wrist osteoarthritis. Your participation may lead to a better understanding of which treatment of wrist osteoarthritis gives the best results in terms of hand and wrist function.

Confidentiality

We need to save and process personal data about you, such as date of birth, gender, questions about employment and information about health. We apply current privacy legislation to all processing of personal data. You will be assigned a special series of numbers (code). The code, together with your social security number and name, forms a code key. Only the undersigned, who are responsible for study, are recipients of the data and who have access to the code key and thus can link the information to you. The code key that is linked to social security number and name will be locked in a safe that only the undersigned have access to. We may share your personal data with a third party, provided that we are required to do so by law. However, we will not transfer your data to a country outside the EU. Your information will be saved as long as there is a purpose for the processing. When this purpose no longer exists, your personal data will be handled in accordance with the legal rules that apply to archiving.

General Data Protection Regulation (GDPR)

Region Skåne, organization number 232100–0255, is responsible for personal data according to the Data Protection Regulation for the processing of personal data. If you agree to participate in the study, you also consent to personal data processing. The data collected in the study is processed and disclosed for statistical analysis only in the coded form described above. A summary of the results of the study will be published in a medical journal, but individuals cannot be identified in such a report.

You have the right to receive information about how your personal data is processed free of charge. You have the right to contact us if you want information about the data we have about you, to request correction, transfer or to request that we restrict the processing, to object or request deletion of your information. The easiest way to do this is to contact us at: Region Skåne, Skåne University Hospital Malmö, Sara Larsson, physiotherapist, VO Specialized surgery, Hand surgery, SE-205 02 Malmö, Sweden. You can also reach our Data Protection Officer at: Data Protection Officer, Region Skåne, SE-291 89 Kristianstad, Sweden. E-mail: region@skane.se. If you are not satisfied with how your personal data has been processed or

feel that your data has been incorrectly handled, you have the opportunity to file a complaint with the supervisory authority Datainspektionen.

Participation and discontinuation of the study
Your participation in this study is completely voluntary and you can discontinue participation at any time without giving any reason and without prejudice to your future treatment. Participation does not incur any additional costs.

Insurance
The patient insurance applies in the study in the same way as for all other treatment in public healthcare.

Additional information

If you want further information, have any questions specifically about the research study, your rights as a study participant or if any other inconvenience should occur, you are welcome to contact the responsible physiotherapist by phone: +46 40-33 67 06, or email: sara.larsson.5408@med.lu.se. You can also contact the responsible researcher Elisabeth Brogren by phone: +46 40 33 1723.

Sara Larsson
Physiotherapist, MSc

Lars B. Dahlin
Professor, MD, PhD

Elisabeth Brogren
Associate professor, MD, PhD

Elisabeth Ekstrand
Physiotherapist, PhD

All at Region Skåne, Skåne University Hospital Malmö, VO Specialized Surgery, Hand Surgery, SE- 205 02 Malmö, Sweden.

Region Skåne, Skåne University hospital Malmö, Sara Larsson, cert. Physioterapist, Handsurgery Rehab, SE-205 02 Malmö, Sweden. +46 40 33 6706

Written consent to participate in the study

Investigation of how neuromuscular training affects wrist function in people with wrist osteoarthritis.

I have been informed in writing about the above study and offered oral information. I have had the opportunity to ask questions about the study and have been given enough time to decide that I can participate in check-ups during and after the treatment period.

I agree; to participate in the study and feel that participation is completely voluntary, that personal data is collected and processed as described in the patient information. Upon written request, I can find out what personal data has been registered about me.

I agree that the measurements made before and after the treatment period are analyzed and used for research in the manner and for the purpose described in the patient information.

I have received a copy of the patient information and written consent.

Date (dated by the patient)

Patient's signature

Name clarification (subtitled)

I confirm that I have explained the design and purpose of the study to participants in the study and that he/she has had the opportunity to ask questions.

Date

Physiotherapist's signature

Name clarification (subtitled)
